# *In Vivo* Evaluation of the Combined Anticancer Effects of Cisplatin and SAHA in Nonsmall Cell Lung Carcinoma Using [^18^F]FAHA and [^18^F]FDG PET/CT Imaging

**DOI:** 10.1155/2021/6660358

**Published:** 2021-03-31

**Authors:** Skye Hsin-Hsien Yeh, Ming Hsien Lin, I. I. Leo Garcia Flores, Uday Mukhopadhyay, Danial Young, Kazuma Ogawa, Jeong-Hwan Jeong, William Tong, Juri G. Gelovani, Nobuyoshi Fukumitsu

**Affiliations:** ^1^Brain Research Center, National Yang Ming Chaio Tung University, Taipei, Taiwan; ^2^Department of Nuclear Medicine, Camillian Saint Mary's Hospital Luodong, Yilan, Taiwan; ^3^Department of Nuclear Medicine, Cheng Hsin General Hospital, Taipei, Taiwan; ^4^Radiomedix, Inc., Houston, USA; ^5^Cyclotron Research Facility, Center for Advanced Biomedical Imaging, U.T.M.D. Anderson Cancer Center, Houston, USA; ^6^Institute for Frontier Science Initiative, Kanazawa University, Kanazawa, Japan; ^7^Department of Nuclear Medicine, Chonbuk National University Medical School & Hospital, Republic of Korea; ^8^Oncology, Neurosurgery, OBGYN, Biomedical Engineering, Wayne State University, Detroit, USA; ^9^Department of Radiation Oncology, Kobe Proton Center, Kobe, Japan

## Abstract

Combining standard drugs with low doses of histone deacetylase inhibitors (HDACIs) is a promising strategy to increase the efficacy of chemotherapy. The ability of well-tolerated doses of HDACIs that act as chemosensitizers for platinum-based chemotherapeutics has recently been proven in many types and stages of cancer *in vitro* and *in vivo*. Detection of changes in HDAC activity/expression may provide important prognostic and predictive information and influence treatment decision-making. Use of [^18^F] FAHA, a HDAC IIa-specific radionuclide, for molecular imaging may enable longitudinal, noninvasive assessment of HDAC activity/expression in metastatic cancer. We evaluated the synergistic anticancer effects of cisplatin and the histone deacetylase inhibitor suberoylanilide hydroxamic acid (SAHA) in xenograft models of nonsmall cell lung cancer (NSCLC) using [^18^F] FAHA and [^18^F] FDG PET/CT imaging. Cisplatin alone significantly increased [^18^F] FAHA accumulation and reduced [^18^F] FDG accumulation in H441 and PC14 xenografts; coadministration of cisplatin and SAHA resulted in the opposite effects. Immunochemical staining for acetyl-histone H3 confirmed the PET/CT imaging findings. Moreover, SAHA had a more significant effect on the acetylome in PC14 (*EGFR* exon 19 deletion mutation) xenografts than H441 (wild-type *EGFR* and *KRAS* codon 12 mutant) xenografts. In conclusion, [^18^F] FAHA enables quantitative visualization of HDAC activity/expression *in vivo*, thus, may represent a clinically useful, noninvasive tool for the management of patients who may benefit from synergistic anticancer therapy.

## 1. Introduction

Lung cancer is the most common cancer and the leading cause of cancer-related deaths worldwide and is more common in developing countries [1]. The majority of cases of lung cancer are nonsmall cell lung cancer (NSCLC), which includes squamous cell carcinoma, adenocarcinoma, and large cell carcinoma [1]. The current classification system for advanced NSCLC includes several histological and molecular subtypes and is a vital component of therapeutic decision-making [2, 3].

Platinum-based agents such as cisplatin are highly active cytotoxic drugs used to treat NSCLC and represent an essential component of neoadjuvant, adjuvant, and palliative chemotherapy regimens [4–7]. Extensive research has demonstrated that changes in various aspects of the use of cisplatin, such as the administration schedule and methods and frequency of monitoring toxicity, have incrementally improved the outcomes and quality of life of patients with NSCLC [5, 8]. Cisplatin is thought to activate DNA damage recognition proteins that transmit DNA damage signals to downstream signaling cascades that involve p53, MAPK, and p73, which ultimately induce apoptosis [9–11].

Several key oncogenic events have been identified in NSCLC. The incidence of epidermal growth factor receptor (*EGFR*) mutations in the Caucasian population is approximately 10%, but is higher among never-smokers, patients with adenocarcinoma, females, and individuals from East Asia [12]. Moreover, the *EML4-ALK* fusion gene is present in approximately 4% of lung tumors and encountered more frequently in the tumors of never-smokers, younger patients, and patients with adenocarcinoma [12]. Thus, only a small proportion of patients with advanced NSCLC are candidates for existing molecular-targeted therapies. For the 85–90% of patients with NSCLC who do not have mutations associated with drug sensitivity (i.e., in genes targeted by EGFR kinase inhibitors), platinum-based chemotherapy remains the standard first-line chemotherapy [4].

The roles of the members of the HDAC family have recently been elucidated in several human malignancies [13]. Histone acetylation and deacetylation of the lysine residues within histone tails occur as a dynamic, reversible process catalyzed by two classes of enzymes, histone acetyltransferase (HAT), and histone deacetylase (HDAC). In general, histone acetylation correlates with transcriptional activation and histone deacetylation correlates with transcriptional repression. Histone acetylation, one of the first epigenetic mechanisms of transcriptional regulation to be studied, is involved in numerous, diverse cellular processes including cell-cycle progression, DNA repair, and gene silencing. Additionally, disruption of the balance between histone acetylation and deacetylation has been implicated as a causative factor in tumor cell proliferation, migration, angiogenesis, differentiation, invasion, and metastasis [14–16].

HDAC inhibitors (HDACIs) are pharmacological compounds with diverse chemical structures that induce hyperacetylation of nuclear histones, weaken histone-DNA interactions, and consequently increase the accessibility of DNA. Several HDACIs have attracted clinical attention [17–19], including suberoylanilide hydroxamic acid (SAHA, Zolinza) [20], depsipeptide (Romidepsin) [21], and panobinostat (LBH589) [22].

SAHA is a potent, reversible pan-HDAC inhibitor. SAHA inhibits both class I and class II HDACs and, thus, alters gene transcription and induces cell cycle arrest and/or apoptosis in a wide variety of transformed cells [23]. SAHA has been clinically approved for the treatment of cutaneous T cell lymphoma [24] and has been shown to exert antitumor activity in other solid tumors, including NSCLC [25–28], breast cancer [29–31], and ovarian cancer [32, 33].

However, HDACIs may block the DNA damage responses induced by cisplatin-mediated toxicity [34, 35]. Through detailed knowledge of the mechanisms underlying the sensitivity of tumors to cisplatin and HDACIs exists, the effects of altered HDAC activity/expression on combination therapy are poorly understood. Therefore, a reliable and quantitative biomarker for HDAC activity is urgently required.

We previously developed 6-[^18^F]fluoroacetamido)–1–hexanoicanilide ([^18^F]FAHA) as a potential PET imaging agent and highly selective radiotracer for quantitative imaging of HDAC class IIa enzyme expression and activity *in vivo* using PET/CT/(MRI) [36, 37]. More recently, we demonstrated that [^18^F] FAHA PET could be used to monitor alterations in HDAC activity/expression in a rat model of chemotherapy-induced neurotoxicity in the brain [38].

Here, we aimed to assess the efficacy of PET/CT using [^18^F] FAHA to image HDAC class IIa activity/expression in a mouse model of NSCLC. We noninvasively monitored the effects of cisplatin in the presence and absence of SAHA on HDAC class IIa activity in H441 (wild-type *EGFR* and *KRAS* codon 12 mutant) and PC14 (*EGFR* exon 19 deletion mutation) NSCLC xenograft tumors. Furthermore, we investigated whether the responses to cisplatin and cisplatin/SAHA were related to the presence of an *EGFR* mutation.

## 2. Materials and Methods

### 2.1. Animals

Eight-week-old male athymic nude mice (*n* = 24, Charles River Laboratories, Middlesex, MA, USA) were used in all studies. The animals were housed at 25°C under a 12 h light/dark cycle and had free access to a standard pellet diet (Lab Diet, Richmond, IN, USA) and tap water. All animal protocols were approved by the Institutional Animal Care and Use Committee of UT MD Anderson Cancer Center (IACUC No. 03-05-01832).

### 2.2. Tumor Xenografts

The NSCLC cell lines H441 (WT *EGFR*, sensitive to cisplatin) and PC14 (*EGFR* exon 19 deletion mutation, resistant to cisplatin) were cultured in flasks in DMEM/F-12 medium supplemented with 10% FBS and antibiotics at 37°C in a humidified atmosphere with 5% CO_2_. Subcutaneous (*s.c.*) H441 and PC14 tumor xenografts were established in the opposite shoulder regions of *nu/nu* mice (*n* = 8 mice per pair of cell lines and per experimental condition) to facilitate direct comparisons of [^18^F] FAHA and [^18^F] FDG accumulation in tumors expressing WT and mutant *EGFR*. When the tumors reached 5–8 mm in diameter, the animals were used for *in vivo* imaging studies.

### 2.3. Study Design and Drug Administration

**Figure 1:**
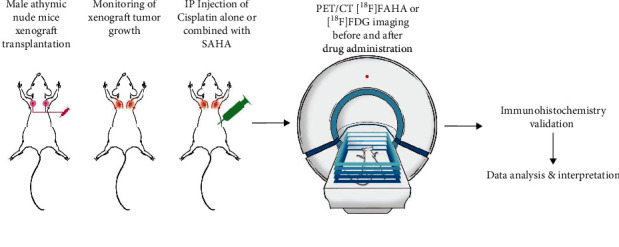
Schematic representation of the study protocol. Animals bearing tumor xenografts were divided to three groups and received intraperitoneal injections of 2 mg/kg cisplatin (Group A), 4 mg/kg cisplatin (Group B), or 4 mg/kg cisplatin with 300 mg/kg SAHA (Group C) (see Materials and Methods for details). PET/CT imaging was performed before and after drug administration. The tumors were excised for IHC after PET/CT imaging.

The mice (*n* = 18–24) were divided into three groups (6-8 mice each). Group A were intraperitoneally (*i.p*.) injected twice with 0.1 mL of cisplatin in saline at 2 mg/kg cisplatin; Group B were *i.p.* injected twice with 0.1 mL cisplatin in saline at 4 mg/kg cisplatin; Group C were *i.p.* injected twice with 0.1 mL of cisplatin in saline at 4 mg/kg cisplatin and injected four times with 300 mg/kg SAHA (10% DMSO in 0.1 mL saline). Two sets of two SAHA injections followed by cisplatin injections at 12 h intervals were given over 5 days between the first and second [^18^F] FAHA and [^18^F] FDG PET scans (Figure 1).

### 2.4. Radiosynthesis

Radiosynthesis of [^18^F] FAHA was performed as described in our previous study [37]. The decay corrected radiochemical yield was 20%, and specific activity was >2 GBq/*μ*mol at the end of synthesis. The overall radiochemical purity was >99%. [^18^F] FDG was purchased from Cyclotope Inc. (Houston, TX, USA), and the specific activity was estimated as >74 GBq/*μ*mol.

### 2.5. PET/CT Imaging

[^18^F] FAHA (day 1) and [^18^F] FDG (day 2) PET imaging studies were performed on consecutive days and repeated 1 week later (days 8 and 9) using an INVEON PET/CT scanner (Siemens Preclinical Solutions, Knoxville, TN, USA). Food was removed the night before the [^18^F] FDG PET study. All mice (*n* = 6 − 8/group, total = 18 − 24) were anesthetized with isoflurane (2% in oxygen). The animals were positioned inside the gantry of the scanner, [^18^F] FAHA (7.4 MBq in 100 *μ*L of saline) was administered intravenously as a slow bolus over 30 sec, and dynamic [^18^F] FAHA PET images were acquired over 30 min. [^18^F] FDG (7.4 MBq in 100 *μ*L of saline) was intravenously administered, and 45 min later, static [^18^F] FDG PET images were acquired over 10 min.

PET images were reconstructed using the two-dimensional ordered subsets expectation maximization algorithm. PET and CT image fusion and image analyses were performed using Inveon Research Workplace software (Siemens Preclinical Solutions, Knoxville, TN, USA). The CT imaging parameters were X-ray voltage, 80 kVp; anode current, 500 *μ*A; and exposure time, 300–350 milliseconds for each of the 360 rotational steps. Images were reconstructed using the Shepp Logan algorithm.

### 2.6. Imaging Data Analysis

A region of interest (ROI) was manually drawn on the axial PET/CT coregistration images of the tumors acquired 20–30 min after injection of [^18^F] FAHA or 40 min after injection of [^18^F]FDG. Dynamic [^18^F] FAHA or static [^18^F] FDG PET images were summed using a rigid transformation algorithm and normalized mutual method after running a reslicing process (PMOD Technologies Ltd., Zurich, Switzerland). The regional radioactivity concentrations (KBq/mL) of [^18^F] FAHA or [^18^F] FDG PET were estimated from the maximum pixel values within each ROI and expressed as a percentage of injected dose/tissue g (%ID/g).

The posttherapy radioactive accumulation rate was calculated using:

(1) $$\begin{array}{c} \text{Post}‐\text{Tx radioactive accumulation rate}=\frac{{\text{Suv}}_{\max}\text{ of Posttherapy}}{{\text{Suv}}_{\max}\text{ of Pretherapy}}-1\times 100\%, \end{array}$$

where SUV_mean_ is the maximum standardized uptake value, and drug inhibition rate is the percentage increase or reduction in accumulation of the PET radiotracer based on the volume accumulated in the tumor.

The SAHA effect rate between Group B and Group C was calculated using:

(2) $$\begin{array}{c} \text{SAHA effect rate}=\frac{{\text{Suv}}_{\max}\text{ of Cisplatin}}{{\text{Suv}}_{\max}\text{ of Cisplatin with SAHA}}-1\times 100\%, \end{array}$$

where SUV_mean_ is the maximum standardized uptake value, and the SAHA effect rate is the percentage increase or reduction in accumulation of the PET radiotracer based on the volume accumulated in the tumor.

### 2.7. Pharmacokinetic Modeling

#### 2.7.1. Simplified Graphic Analysis: Patlak Plot

The tumor and thoracic aorta time-activity data from the dynamic [^18^F] FAHA PET scans were used for the Patlak plot analyses [36, 38], as follows:

(3) $$\begin{array}{c} \left(R\left(t\right)\right)/\left(C\left(t\right)\right)=\text{Ki}\int_{0}^{t}C\left(t\right)dt/C\left(t\right)+V_{0}. \end{array}$$

where *t* is time, *R* (*t*) is the mean count of the tumor, *C* (*t*) is the mean count of the blood, Ki is the clearance that determines the rate of entry into the tumor, and *V*_0_ is the distribution volume.

The time between injection and the start of the linear phase in the Patlak plot was 4–6 min. Based on data from the start of the linear phase, an accurate linear fit was observed from 6–8 min up to 18–20 min. The slope of the Patlak plot represents the influx rate constant Ki.

### 2.8. Immunohistochemistry

After imaging, the mice were humanely euthanized, and the tumors were excised for immunohistochemical analysis (IHC) of acetyl-histone H3 (AH3). Paraffin-embedded sections (5 *μ*m-thick) were incubated in antigen retrieval solution (10 mM citrate buffer, pH 6.0) at 100°C for 10 min, washed, incubated in 3% hydrogen peroxidase solution for 15 min at room temperature, and then placed in blocking solution for 60 min at room temperature. Then, the sections were incubated with a primary acetyl-histone A3 (Lys9) antibody (Cell Signaling Technology, Danvers, MA, USA) at 1 : 50 dilution in blocking solution overnight at 4°C, followed by secondary biotinylated horse anti-mouse IgG (Vector Laboratories, Inc., Burlingame, CA, USA), and developed via avidin-peroxidase conjugation and the chromophore 3′3-diaminobenzidine using a Vectastain ELITE kit (Vector Laboratories, Burlingame, CA, USA) according to the manufacturer's protocol. The tissue sections were either counterstained with hematoxylin or not counterstained for densitometric analysis of the intensity of AH3 immunostaining.

Microscopic evaluation of the immunostained sections was performed using a BX51 microscope equipped with a DP71 digital camera (Olympus, Tokyo, Japan). Protein expression was semiquantitatively assessed based on the number of cells showing nuclear expression of AH3 in five nonoverlapping ×100 microscopic fields as: 0 = absent, less than 5% immunopositive cells; 1 = rare, 10–20% immunopositive cells; 2 = mild, 20–40% mildly or moderately positive cells; 3 = moderate, 40–60% moderately or strongly positive cells; or 4 = strong, more than 80% strongly positive cells per field of view. The percentage score for each tumor was calculated as follows: actual rating × 100/maximal score (i.e., a rating value of 4).

(4) $$\begin{array}{c} \text{Percentage of positive signal}=\frac{\text{Sum of score of the group}}{\text{Number of case}\times \text{maximal score }4}\times 100\%. \end{array}$$

### 2.9. Statistical Analysis

Values are expressed as minimum to maximum and average values. The Ki value of [^18^F]FAHA and %ID/g of [^18^F] FDG before and after drug administration (cisplatin alone or combined with SAHA) was investigated using paired *t*-tests. Significance was defined as *p* < 0.05.

## 3. Results

### 3.1. Cisplatin Increases the [^18^F]FAHA PET/CT Signal for HDAC IIa in NSCLC Xenograft Tumors and Coadministration of SAHA Abolishes This Effect

**Figure 2:**
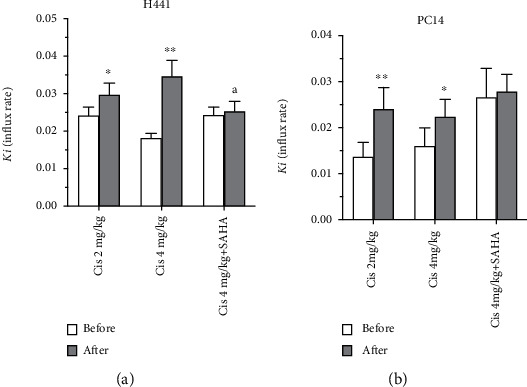
Comparison of tumor [^18^F]FAHA Ki values, based on graphical analyses before and after drug administration. [^18^F]FAHA Ki values significantly increased in the H441 and PC14 xenografts after administration of cisplatin in Groups A (2 mg/kg cisplatin) and B (4 mg/kg cisplatin), but not in Group C (4 mg/kg cisplatin +300 mg/kg SAHA). Data are mean ± SEM. Different superscript letters indicate significant differences (^∗^*p* < 0.05, ^∗∗^*p* < 0.005 compared to the Ki values before drug administration; ^a^*p* < 0.05, Group C vs. Group B).

**Figure 3:**
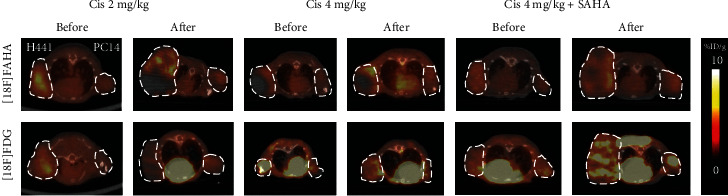
Axial tumor [^18^F]FAHA (upper panel) and [^18^F] FDG (lower panel) imaging before and after drug administration. [^18^F]FAHA PET/CT images were summed over 10 min (20–30 min after [^18^F]FAHA injection). Cisplatin significantly increased [^18^F]FAHA radioactivity in H441 and PC14 xenograft tumors, and this effect was blocked by SAHA. [^18^F] FDG imaging at 45–55 min after injection. Tumor [^18^F] FDG accumulation decreased after administration of cisplatin, but increased after administration of cisplatin with SAHA.

The Ki value for [^18^F]FAHA in H441 tumor xenografts significantly increased after administration of cisplatin in both Groups A and B (Figure 2(a), *p* < 0.05 and *p* < 0.005, respectively), but not after administration of cisplatin with SAHA in Group C (Figures 3 upper panel and 2(a)). Coadministration of SAHA significantly blocked the cisplatin-induced increase in the Ki value for [^18^F]FAHA in H441 tumors (Figure 2(a), *p* < 0.05). Similar quantitative trends, though at a lower magnitude, were observed in the PC14 xenografts (Figures 3 lower panel and 2(b), *p* < 0.005 and *p* < 0.05, respectively).

The changes in the [^18^F]FAHA *Ki* values in H441 or PC14 tumors in Groups A and B were not dependent on the dose of cisplatin.

### 3.2. Cisplatin Reduces the [^18^F] FDG PET/CT Signal for Glucose Metabolism and Coadministration of SAHA Increases [^18^F] FDG Accumulation

**Figure 4:**
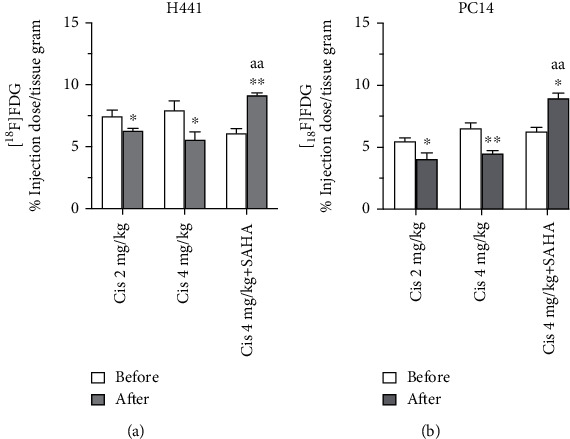
Comparison of [^18^F] FDG accumulation in xenograft tumors before and after drug administration. Accumulation of [^18^F] FDG significantly decreased after administration of cisplatin in Groups A and B, but increased after administration of cisplatin + SAHA in Group C. Data are mean ± SEM. Different superscript letters indicate significant differences (^∗^*p* < 0.05, ^∗∗^*p* < 0.01 compared to the [^18^F] FDG SUV before drug administration; ^aa^*p* < 0.005, Group C vs. Group B).

Accumulation of [^18^F] FDG radioactivity in H441 tumor xenografts decreased significantly after administration of cisplatin in Groups A and B (Figures 3 lower panel and 4(a), *p* < 0.05). However, in Group C, the accumulation of [^18^F] FDG in H441 tumors dramatically increased after administration of cisplatin with SAHA (*p* < 0.005, Figure 4(a)). Compared to cisplatin alone (Group B), the combination of cisplatin with SAHA (Group C) significantly increased [^18^F] FDG in H441 tumors (*p* < 0.005, Figure 4(a)). Similar trends were observed in PC14 tumors (Figures 3 lower panel and 4(b)).

Cisplatin did not have a dose-dependent effect on [^18^F] FDG accumulation in H441 or PC14 tumors between Groups A and B. The SUV uptake data for [^18^F]FAHA or [^18^F] FDG in H441 and PC14 tumors are listed in Table 1.

### 3.3. H441 And PC14 Tumor Xenografts Exhibit Different Responses to SAHA

**Figure 5:**
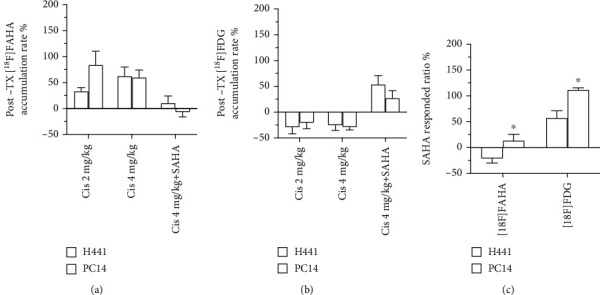
Comparison of [^18^F]FAHA or [^18^F] FDG accumulation in H441 and PC14 xenograft tumors postdrug administration. There were no significant differences in the increase ratios for [^18^F]FAHA (a) or [^18^F] FDG (b) between H441 and PC14 xenograft tumors. (c) After treatment with cisplatin + SAHA, the [^18^F]FAHA and [^18^F] FDG ratios were significantly higher in PC14 xenograft tumors than H441 xenograft tumors. Data are mean ± SEM; ^∗^*p* < 0.05, H441 vs. PC14.

Compared to the H441 tumors, PC14 xenografts exhibited relatively higher cisplatin-induced accumulation rates, as indicated by higher [^18^F]FAHA Ki values and lower [^18^F] FDG uptake after injection with cisplatin alone. However, the differences between H441 and PC41 tumors were not statistically significant (Figures 5(a) and 5(b)).

When comparing the accumulation of [^18^F]FAHA or [^18^F] FDG radioactivity between Group B and Group C (cisplatin alone vs. cisplatin/SAHA), PC14 tumors exhibited significantly higher ratios for both [^18^F]FAHA (*p* < 0.05) and [^18^F] FDG (*p* < 0.05) compared to H441 tumor cells (Figure 5(c)).

### 3.4. SAHA Increases Histone H3 Acetylation in NSCLC

**Figure 6:**
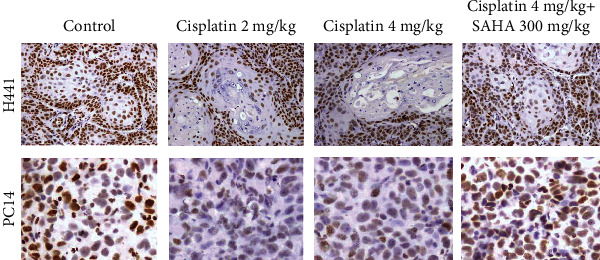
Immunohistochemical staining for AH3 in xenograft tumors. In H441 xenograft tumors, AH3 immunoreactivity was lower in Group A (2 mg/kg cisplatin) and Group B (4 mg/kg cisplatin) than the control group and was almost equal in Group C (4 mg/kg+300 mg/kg SAHA) and the control group. Similar trends were observed in PC14 tumors; however, Group C exhibited a significantly higher increase in AH3 immunoactivity than Group B. Scale bar, 100 *μ*m.

IHC demonstrated that AH3 immunoactivity significantly decreased in H441 tumors after administration of cisplatin alone or cisplatin combined with SAHA in Groups A, B, and C compared to control mice (*p* < 0.05, Figures 6 upper panel and 7(a)). AH3 immunoactivity was not markedly different between Groups B and C.

**Figure 7:**
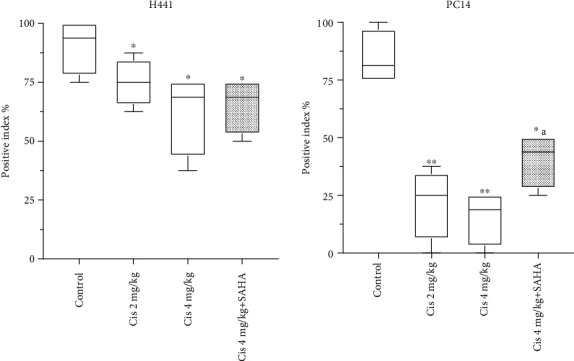
Quantification of AH3 immunoreactivity in the tumors (based on the data in Figure 5). Data are mean and mix/max. Values; different superscript letters indicate significant differences (^∗^*p* < 0.05, ^∗∗^*p* < 0.01 compared to controls; ^a^*p* < 0.05, Group C vs. Group B).

In the PC14 tumor sections, cisplatin significantly reduced AH3 immunoactivity in Groups A (*p* < 0.005) and B (*p* < 0.005; Figures 6 lower panel and 7(b)). However, the combination of SAHA with cisplatin (Group C) reversed the cisplatin-induced reduction in AH3 immunoactivity compared to mice injected with cisplatin alone (Group B; *p* < 0.05, Figure 7(b)).

## 4. Discussion

Histone deacetylation plays a critical role in the regulation of various cellular processes, including nucleosome assembly, chromatin folding, DNA damage repair, and transcription. Furthermore, aberrant histone deacetylation has been implicated in the etiology of a variety of diseases, as well as the adverse effects of chemical exposure. The synergistic anticancer effects of cisplatin and SAHA have been demonstrated in a variety of cancer cell lines and animal tumor xenograft models, as described in the Introduction. However, little is known about the direct effects of epigenetic regulators, or the subsequent targeted effects, in NSCLC. This is the first *in vivo* PET/CT imaging assessment of the synergistic anticancer effects of cisplatin and SAHA on HDAC IIa activity/expression and glucose metabolism in an animal model of NSCLC. Cisplatin increased the Ki value of [^18^F]FAHA in the tumors and decreased [^18^F] FDG uptake. These results demonstrate cisplatin promotes excessive HDAC deacetylation and suppresses DNA transcription in NSCLC tumor cells. Additionally, IHC confirmed the results of the PET/CT imaging, as cisplatin led to overexpression of the HDAC deacetylase AH3 in tumor cells, which would subsequently decrease DNA transcription.

Consistent with our results, Wang et al. (2017) reported that pretreatment with cisplatin activated the HDAC Tribbles Pseudokinase 1- (TRIB1-) interacting protein. TRIB1 and HDACs play crucial roles in cisplatin-induced enrichment of cancer stem cells (CSCs) and drug resistance in NSCLC, and patients with high levels of TRIB1 had a significantly poorer response to cisplatin and a poorer prognosis [39]. Furthermore, cisplatin and SAHA suppressed the viability and growth of NSCLC tumor cells *in vitro* and *in vivo* [39].

Moreover, Sun et al. (2019) reported that cotreatment with S11—which simultaneously inhibits HDACs and P-glycoprotein (P-gp)—and cisplatin suppressed colony formation and blocked the migration of cisplatin-resistant NSCLC cells [40]. Subsequently, the combination of SAHA with cisplatin was shown to induce miR-149 expression, which directly targets the excision repair cross-complementation group (ERCC1). Inhibition of ERCC1 expression correlated positively with DNA repair capacity, thus, miR-149 and ERCC1 may represent a target to increase the sensitivity of tumor cells to cisplatin [41, 42]. Combined administration of SAHA and cisplatin also induced apoptosis, promoted cell cycle arrest, and increased the levels of acetylated histone H3 and *α*-tubulin in a xenograft model of small cell lung cancer (SCLC) in nude mice [43]. Additionally, previous reports demonstrated various HDACIs exert synergistic antitumor effects with fluoropyrimidines in several tumor types, including breast cancer, colorectal cancer [44–47], and head and neck squamous cell carcinoma. Overall, these studies indicate inhibition of HDACs sensitizes tumor cells to cisplatin *in vitro* and *in vivo* and that HDACIs may provide an approach to block the development of cisplatin resistance.

[^18^F]fluoro-2-deoxy-D-glucose positron emission tomography ([^18^F] FDG PET) is widely used to detect and stage tumors [48]. Thus, we used [^18^F] FDG PET to monitor the therapeutic effects of cisplatin and SAHA in NSCLC *in vivo*. Cisplatin-mediated cancer therapy decreases [^18^F] FDG PET [49–51] signals, which represent the hallmarks of altered energy metabolism in cancer. Mono-chemotherapy with HDACIs also reduces [^18^F] FDG PET [51] signals.

HDACIs promote CSC expansion by reprogramming differentiated cancer cells into stem-like cells that exhibit enhanced Pentose phosphate pathway (PPP) metabolism. PPP plays a substantial role in the production of cellular NADPH, which is required for fatty acid synthesis and intracellular ROS detoxification [52]. Interestingly, we found that cisplatin alone significantly reduced [^18^F] FDG uptake in the NSCLC xenografts. However, this effect was reversed by coadministration of SAHA, consistent with our previous findings in a rat model of cisplatin-induced neurotoxicity [38]. Therefore, the significant enhancement in the [^18^F] FDG PET signals in NSCLC tumors implies that the combination of cisplatin and SAHA may—at least partially—enrich CSCs, since glucose is one of the main energy sources for both CSCs and differentiated tumor cells [53].

Accumulating evidence indicates HDACIs modulate the epigenetic regulation of CSCs, specifically the CSC subpopulation, in solid cancers [54]. HDACIs have been suggested to modulate stemness and enable tumor cells to overcome drug resistance [55, 56]. Our results demonstrate that the changes in HDAC activity/expression and metabolic adaptation of CSCs included by combined treatment with chemotherapy and HDACIs could be monitored *in vivo* using coupled [^18^F] FDG and [^18^F]FAHA PET/CT imaging.

Cisplatin-based chemotherapy remains the first-line strategy for wild-type *EGFR* in NSCLC; however, cisplatin often becomes ineffective as most tumors acquire drug resistance over time. Cell lines expressing mutant *EGFR* are mostly resistant to cisplatin, and *KRAS* mutant cell lines exhibit varied sensitivity to cisplatin, depending on E-cadherin mRNA expression [57]. In the present study, we conducted follow-up [^18^F] FDG PET/CT scans after completion of treatment, which is a standardized imaging procedure for monitoring the response to therapy [58]. [^18^F] FDG uptake is proportional to the metabolic rate of viable tumor cells, which have a higher demand for glucose than normal cells [59].

Similarly to a previous report [57], cisplatin alone led to a relatively poor cisplatin-inhibition rate (lower [^18^F] FDG reduction) and moderate HDAC activity (higher [^18^F]FAHA increase) in PC14 xenografts (*EGFR* exon 19 deletion mutant) than the H441 xenografts (wild-type *EGFR* and *KRAS* codon 12 mutant). As PC14 xenografts exhibited moderate HDAC activity (reduced [^18^F]FAHA accumulation) and increased energy demand (increased [^18^F] FDG uptake), combination therapy with SAHA may lead to CSC expansion and promote a transition to stem-like cells with altered metabolic adaptation for glucose. The current results also demonstrate that PC14 cells—which are most likely resistant to cisplatin—were more sensitive to SAHA, as revealed by the significantly higher [^18^F]FAHA and [^18^F] FDG signals in PC14 xenografts compared to H441 xenografts [60].

Immunostaining for AH3 confirmed the PET/CT imaging findings, in that cisplatin increased HDAC activity/expression by reducing the expression of AH3, whereas coadministration of SAHA reversed these effects. Moreover, the immunostaining also supported the PET/CT finding that the magnitude of the changes in HDAC activity/expression was greater in the PC14 xenografts than H441 xenografts.

Overall, coadministration of cisplatin and SAHA did not change the Ki values of [^18^F]FAHA in either PC14 or H441 xenografts, although the [^18^F]FAHA Ki values significantly increased after administration of cisplatin alone. Conversely, cisplatin and SAHA coadministration increased [^18^F] FDG accumulation, whereas cisplatin alone reduced [^18^F] FDG accumulation. These results suggest that SAHA blocks excess HDAC deacetylation and would thus inhibit the antitumor effects of cisplatin in NSCLC. In addition, the IHC also supported our hypothesis that SAHA blocks cisplatin-induced decreases in HAT acetylation and increases in HDAC deacetylation in NSCLC.

The effects of HDACIs in cancer have been examined in preclinical and early clinical studies. HDACIs are expected to be used in combination with other anticancer drugs. HDACIs synergistically enhance the anticancer effects of chemotherapy drugs in several tumor types [23, 61–63]. However, HDACIs do not cooperate with anticancer drugs to synergistically inhibit cell proliferation in all tumor types. For example, Chai et al. (2008) reported the HDACIs depsipepside and Trichostatin A enhanced cytarabine-induced inhibition of cell proliferation, but synergistic inhibition of cell proliferation was not observed for other DNA damage inducers—including cisplatin. Chai et al. concluded HDACIs selectively act with nucleoside analogs to inhibit cell proliferation [64]. Differences between the tumor models and anticancer drugs may be one explanation for these discrepancies.

Platinum-based therapy still represents a major therapeutic strategy in several solid tumors, including colorectal, breast, and pancreatic cancer. However, chemo-resistance remains a major unresolved issue. As discussed above, HDACIs modulate gene expression and usually function as sensitizers to act synergistically with chemotherapeutics and molecular targeted agents. Although numerous studies have demonstrated the benefit of coadministration of cisplatin with SAHA as mentioned before, the mechanisms by which HDACIs interact with cisplatin in cancer cells have not yet been elucidated. Furthermore, we have no knowledge of the metabolic adaptations that take place during the transition from normal stem cells to CSCs induced by such cancer therapy, and only a handful of studies have explored the transition of CSCs to differentiated tumor cells [39]. Moreover, additional studies are needed to evaluate the clinical applicability of SAHA or other HDACIs as a component of chemoradiotherapy regimens. Thus, repetitive, noninvasive PET/CT imaging with [^18^F]FAHA may facilitate future preclinical or clinical studies to elucidate the roles of class IIa HDAC enzymes in tumor progression, chemoresistance, and the expansion of CSCs, and may help to optimize therapeutic doses of novel class IIa HDACIs for combined chemoradiotherapy.

## 5. Conclusion

Combining traditional chemotherapy drugs with HDACIs may improve therapeutic efficacy in solid cancers. Molecular imaging using [^18^F] FAHA, a novel HDAC IIa-specific radiotracer, provided unique insight into the location of and quantitative changes in HDAC activity/expression in tumors *in vivo* in response to treatment with cisplatin alone or cisplatin combined with a HDACI. Additional PET imaging may help to determine the mechanistic, therapeutic, and prognostic roles of HDACs in various diseases and enable monitoring of HDAC-targeted therapies. Further clinical and preclinical investigations are necessary to identify the mechanisms by which HDACIs modulate signaling pathways in different tumor types.

## Figures and Tables

**Table 1:** Comparison of [^18^F]FAHA *Ki* values or [^18^F] FDG %ID/g accumulation in xenograft tumors before and after drug administration. Data are mean ± SEM. Different superscript letters indicate significant differences (^∗^*p* < 0.05, ^∗∗^*p* < 0.005 compared to the Ki values before drug administration; ^a^*p* < 0.05, ^aa^*p* < 0.005 Group C vs. Group B).

	Cis 2 mg/kg			Cis 4 mg/kg			Cis 4 mg + SAHA 300 mg/kg		
	Before	After	*p*	Before	After	*p*	Before	After	*p*
[^18^F]FAHA									
H441	0.024 ± 0.006	0.030 ± 0.009	∗	0.018 ± 0.003	0.035 ± 0.012	∗∗	0.024 ± 0.006	0.025 ± 0.008	a
PC14	0.014 ± 0.003	0.027 ± 0.009	∗	0.016 ± 0.004	0.026 ± 0.009	∗	0.027 ± 0.006	0.031 ± 0.008	

[^18^F]FDG									
H441	7.444 ± 1.334	6.293 ± 0.501	∗	7.934 ± 2.030	5.559 ± 1.716	∗	6.094 ± 0.991	9.156 ± 0.468	∗∗aa
PC14	5.474 ± 0.723	4.044 ± 1.359	∗	6.544 ± 1.017	4.504 ± 0.637	∗∗	6.750 ± 1.596	9.289 ± 3.540	∗aa

## Data Availability

All data generated or analyzed during this study are included in this published article.
